# The influences of purple sweet potato anthocyanin on the growth characteristics of human retinal pigment epithelial cells

**DOI:** 10.3402/fnr.v59.27830

**Published:** 2015-06-11

**Authors:** Min Sun, Xiaoling Lu, Lei Hao, Tao Wu, Huanjiao Zhao, Chao Wang

**Affiliations:** Key Laboratory of Food Nutrition and Safety of the Ministry of Education, College of Food Engineering and Biotechnology, Tianjin University of Science & Technology, Tianjin, China

**Keywords:** PSPA, RPE cells, morphology, survival, proliferation

## Abstract

**Background:**

Anthocyanins have been proven to be beneficial to the eyes. However, information is scarce about the effects of purple sweet potato (Ipomoea batatas, L.) anthocyanin (PSPA), a class of anthocyanins derived from purple sweet potato roots, on visual health.

**Objective:**

The aim of this study was to investigate whether PSPA could have influences on the growth characteristics (cellular morphology, survival, and proliferation) of human retinal pigment epithelial (RPE) cells, which perform essential functions for the visual process.

**Methods:**

The RPE cell line D407 was used in the present study. The cytotoxicity of PSPA was assessed by MTT assay. Then, cellular morphology, viability, cell cycle, Ki67expression, and PI3K/MAPK activation of RPE cells treated with PSPA were determined.

**Results:**

PSPA exhibited dose-dependent promotion of RPE cell proliferation at concentrations ranging from 10 to 1,000 µg/ml. RPE cells treated with PSPA demonstrated a predominantly polygonal morphology in a mosaic arrangement, and colony-like cells displayed numerous short apical microvilli and typical ultrastructure. PSPA treatment also resulted in a better platform growing status, statistically higher viability, an increase in the S-phase, and more Ki67+ cells. However, neither pAkt nor pERK were detected in either group.

**Conclusions:**

We found that PSPA maintained high cell viability, boosted DNA synthesis, and preserved a high percentage of continuously cycling cells to promote cell survival and division without changing cell morphology. This paper lays the foundation for further research about the damage-protective activities of PSPA on RPE cells or human vision.

Polyphenolic compounds, the most abundant antioxidants people can get from dietary sources, are considered to be protective of vision health and retinal cells (1–3). PSPA, a class of naturally occurring anthocyanins derived from purple sweet potato storage roots, can be used as food colorant. PSPA has been proved to possess multiple bioactivities (4). Recently, products made from purple sweet potato, rich in anthocyanins, have become popular health foods (5–8). The major components of PSPA are cyanidin acylglucosides and peonidin acylglucosides, and acylated anthocyanins in purple sweet potato can be absorbed directly by rats and humans in intact acylated forms (9–11). Furthermore, anthocyanins can pass the blood–brain barrier and blood–retinal barrier and accumulate in the eyes, as observed *in vivo* (12, 13). However, the potential of PSPA to protect the eyes has not yet been investigated.

The RPE, a monolayer of highly polarized cells strategically situated behind the photoreceptor cells (14), performs essential functions for the survival of retinal neurons and is important for the maintenance of the visual process (15). RPE cells mediate the flow of nutrients, metabolites, and ions between the choroid and the neural retina (16). They also participate in converting and storing retinoid, which is necessary for the visual process (17). Due to their unique functions and position, the RPE cells suffer from oxidative stress (18) and light exposure (19). Moreover, RPE dysfunction leads to many devastating retinal diseases, such as age-related macular degeneration (20). Maintaining the RPE-specific phenotype and sustaining the structural integrity and normal physiological functions of RPE cells is crucial for retinal health. Thus, the effect on human eye health of protecting RPE cells is a relevant topic for research; RPE cells have been used in many *in vitro* studies (21, 22). In one study, it was shown that green tea polyphenols protected RPE cells from UVB damage (23); another study found that blueberry anthocyanin suppressed RPE cell aging and apoptosis and protected them from visible-light-induced damage (24). However, few studies have shown the influences of polyphenols on normal RPE cellular morphology, survival, and proliferation without light or oxidative damage, which might have effects on the resistance of RPE cells to damage.

In view of all these considerations, the purpose of the present study was to explore whether PSPA could influence growth characteristics such as cellular morphology, survival, and proliferation of RPE cells, in order to lay the foundation for damage-protection research and throw new light on the role of PSPA on human eye health.

## Materials and methods

### Materials

The human RPE cell line (D407) was purchased from the Animal Experiment Center of Sun Yat-Sen University (Guangzhou, China). PSPA used in the study was supplied by Huludao Maohua Biology Co., Ltd. (Liaoning, China); the major components of the PSPA by High Performance Liquid Chromatography – Mass Spectrometry (HPLC-MS) analysis were cyanidin acylglucosides and peonidin acylglucosides (>85%). Dulbecco's modified Eagle's Medium (DMEM), penicillin, streptomycin, 0.5% (vol/vol) trypsin/EDTA, and fetal bovine serum (FBS) were purchased from Gibco Life Technologies (Grand Island, NY, USA). MTT (3-[4,5-dimethylthiazol-2-yl]-2,5-diphenyltetrazolium bromide) was acquired from Sigma–Aldrich, Inc. (St. Louis, MO, USA). From Corning Glassworks (Corning, NY, USA), 96-well plates, 6-well plates, and 25 cm^2^ flasks were purchased. A Muse^TM^ Count & Viability Assay Kit, Cell Cycle Kit, Ki67 Proliferation Kit, and PI3K/MAPK Dual Pathway Activation Kit were purchased from Merck Millipore (Billerica, MA, USA).

### Cell culture and PSPA treatment

The RPE cells were grown in whole culture medium, namely, DMEM with 10% FBS and containing a 1% antibiotic mixture of penicillin (100 U/ml) and streptomycin (100 mg/ml). Cells were incubated at 37°C under a humidified 5% CO_2_ atmosphere. When the cells were confluent, they were detached with 0.5% (vol/vol) trypsin/EDTA after a rinse with 0.1 M phosphate-buffered saline.

The PSPA was dissolved in DMEM without the FBS supplement at a concentration of 500 mg/l as a stock solution and stored at −20°C. Before all experiments, the stock solution was sterilized by processing through a 0.1 µm filter, and then it was diluted with DMEM to certain concentrations. Ten percent FBS was added to the PSPA culture medium.

### Evaluation of cytotoxicity of PSPA

RPE cells were seeded in 96-well plates at a concentration of 2×10^5^ cells/ml and allowed to attach for 1 day. The medium was then replaced with 0, 10, 100, 1,000, and 10,000 µg/ml PSPA culture medium. After PSPA treatment for 1 day, post-culture was conducted with whole culture medium for either 1 or 2 more days. MTT assay (25) was used to detect the optical density (OD) values of each group at each time points. Further experiments were processed to evaluate the long-term cytotoxicity of PSPA: namely, the PSPA culture medium was used to resuspend and plate RPE cells, and the treatments were terminated at 1, 2, and 3 days without post-culture.

### Observation of cellular morphology

#### Inverted phase contrast microscopy

RPE cells suspended in whole culture medium and 100 µg/ml PSPA culture medium were seeded on cover slips at a density of 5×10^5^ cells/ml. Specimens cultured with each medium for 2 and 4 days were viewed by phase contrast microscopy without further processing.

#### Scanning electron microscopy

Specimens cultured with whole culture medium and 100 µg/ml PSPA culture medium for 2 days were prepared according to the literature (26). Samples were then examined using Scanning electron microscope (SEM) (SU-1510; Hitachi Ltd., Tokyo, Japan).

#### Transmission electron microscopy

Samples were fixed, dehydrated, and embedded referring to the literature (26). Sections of 50–60 nm thickness were made on an ultramicrotome (Ultracut-R; Leica, Wetzlar, Germany) and were stained using uranyl acetate and alkaline lead citrate for 15 min. Sections were examined and photographed with a transmission electron microscope (TEM) (H-7500, Hitachi, Ltd.).

### Determination of cell growth curve

To obtain growth curves for RPE cultured with and without PSPA, RPE cells suspended in whole culture medium and 100 µg/ml PSPA culture medium were seeded in 96-well plates at a density of 2×10^5^ cells/ml. The cells incubated with each medium for 6 days and OD values were detected by MTT assay at each day.

### Assay of cell viability

The viability assay was performed according to the user's manual, using the Muse^TM^ Cell Analyzer (Merck Millipore); 2,000 events were acquired for each sample. The viable cells and total cells were each counted, and viability was expressed as a percentage of the viable cells.

### Analysis of cell cycle

Cell cycle analyses were performed with the Muse^TM^ Cell Analyzer according to the Muse™ Cell Cycle Kit user's guide, after RPE cells were cultured in whole culture medium and 100 µg/ml PSPA culture medium for 1–3 days. For each sample, 20,000 events were recorded. The results were analyzed using ModFit 3.2 (Verity Software House, Topsham, ME, USA).

### Detection of Ki67 expression

RPE cells were cultured in whole culture medium and 100 µg/ml PSPA culture medium for 1–3 days. The Ki67 proliferation assay was then performed according to the Muse^TM^ Ki67 Proliferation Kit user's guide with the Muse^TM^ Cell Analyzer.

### Examination of PI3K/MAPK expression

RPE cells suspended in whole culture medium and 100 µg/ml PSPA culture medium were cultured for 1–3 days. The Muse™ PI3K/MAPK Dual Pathway Activation Kit was used to assess the activation of both the PI3K and MAPK signaling pathways simultaneously, according to the user's guide.

### Statistical analysis

All experiments were performed in triplicate; results were given in means±standard deviation. One-way ANOVA with Duncan's multiple comparison test were performed with SPSS software, version 18.0 (SPSS Inc., Chicago, IL, USA) for cell cytotoxicity assays. Unpaired Student *t*-tests were used for other assays. Results were considered statistically significant at *p*<0.05.

## Results and discussion

### Cytotoxicity of PSPA on RPE cells

**Fig. 1:**
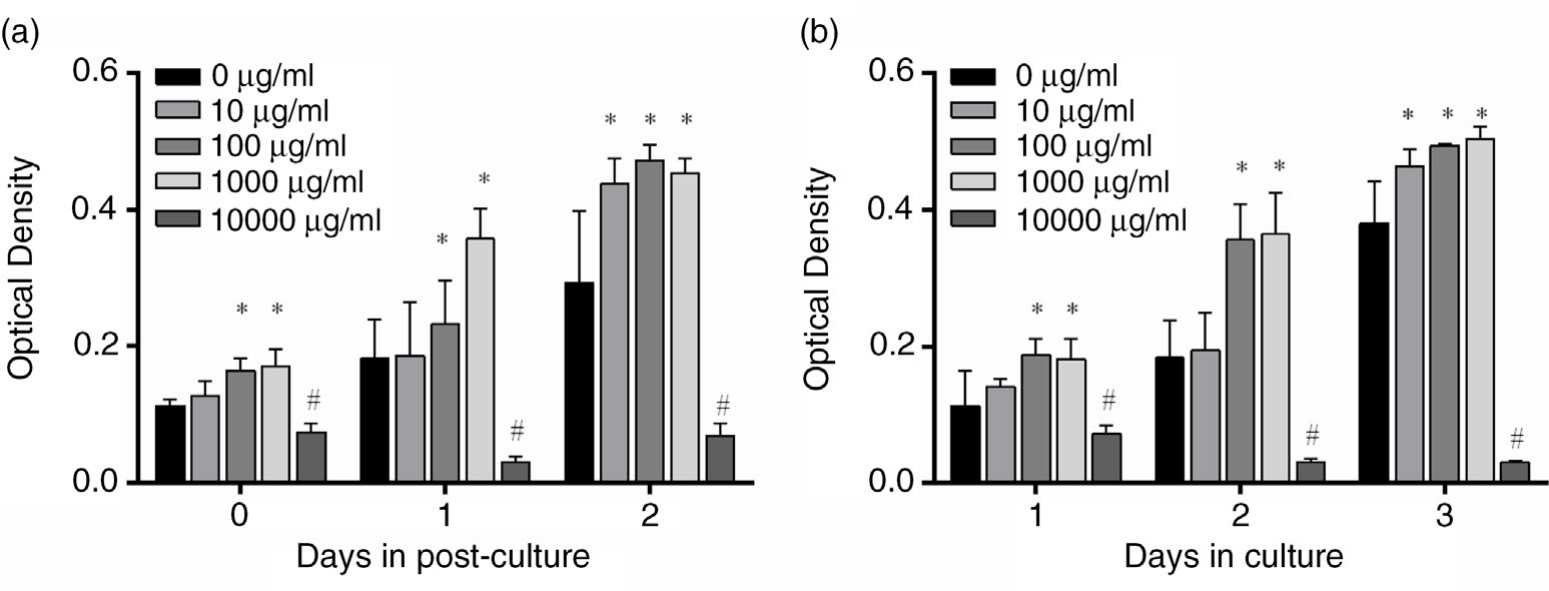
Cytotoxic effect of purple sweet potato anthocyanin (PSPA) on retinal pigment epithelial (RPE) cells. RPE cells (2×10∧5 cells/ml) were incubated with whole culture medium and allowed to attach for 1 day. The cells were then exposed to 0, 10, 100, 1,000, or 10,000 µg/ml PSPA medium for 1 day, and post-culture was conducted with whole culture medium for either another 1 or 2 days. MTT assay was used to detect optical density values for each group on Days 0, 1, and 2 post-culture (a). In (b), PSPA culture medium was used to resuspend and seed RPE cells (2×10^5^ cells/ml), and the treatments were terminated at Days 1, 2, and 3, without post-culture. Values are means, with standard deviations represented by vertical bars (*n*=3). *Mean values were significantly higher; ^#^mean values were significantly lower than 0 µg/ml group, *p*<0.05 (one-way ANOVA).

Two independent experiments were carried out to study the cytotoxicity of PSPA on RPE cells; the results are shown in Fig. 1. A dose-dependent promotion of RPE cell proliferation was observed when the concentrations of PSPA ranged from 10 to 1,000 µg/ml. Compared to the control group, a stimulation of RPE cell proliferation cultured with 10 µg/ml PSPA was only observable when cells were treated with PSPA for 1 day followed by a 2-day incubation, or on the third day of continuous incubation. No differences were found between the effects of PSPA on RPE cells at concentrations of 100 and 1,000 µg/mL; PSPA at both concentrations improved RPE cell proliferation by over 1.3-fold compared to that of the control (Fig. 1a and 1b). At 10,000 µg/ml, the cytotoxicity of PSPA was indicated by a remarkable decrease in OD values. Thus, doses of 100 µg/ml of PSPA were selected for the following study.

### Cell morphology of RPE cells treated with PSPA

The reported epithelioid phenotype of RPE cells with polygonal morphology and colony-like distribution suggested that the cells maintained their specific cell functions (27, 28). We observed the cellular morphology of RPE cells treated with PSPA in order to show whether PSPA could preserve the phenotype of RPE cells.

**Fig. 2:**
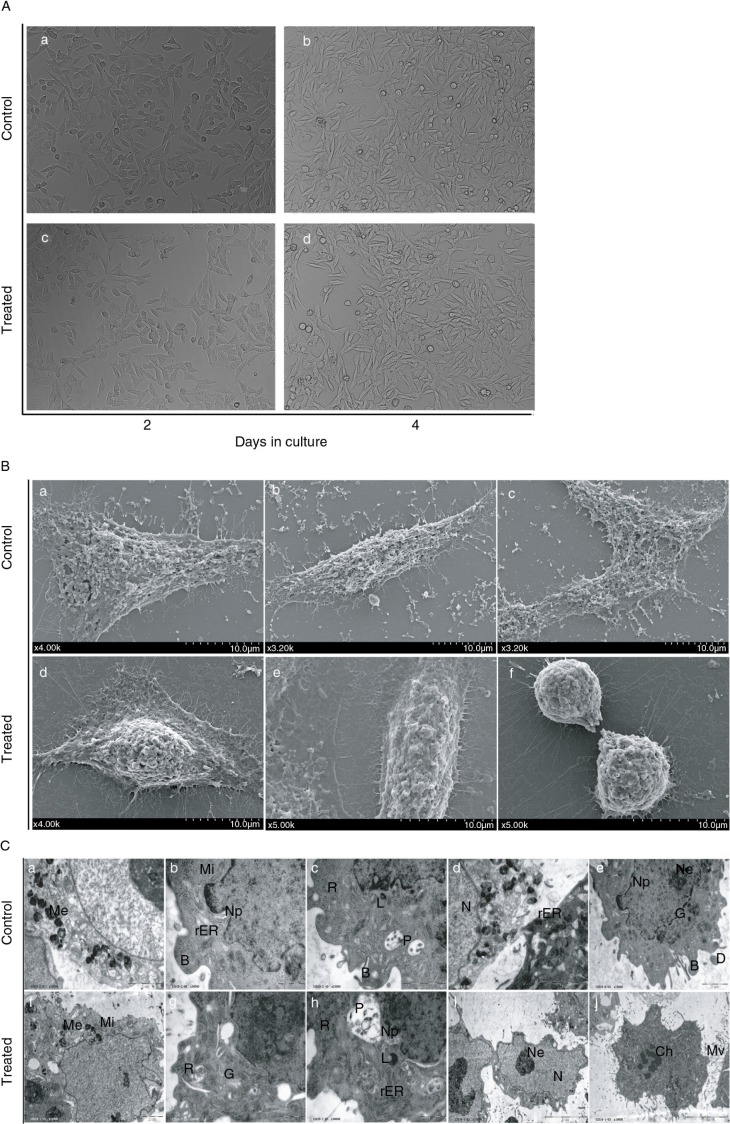
Morphological observation of RPE cells treated with 100 µg/ml PSPA. Fig. 2A, phase-contrast micrographs of RPE cells cultured without PSPA (control) for (a) 2 days and (b) 4 days, and with 100 µg/ml PSPA (treated) for (c) 2 days and (d) 4 days. Scale bar: 20 μm. Fig. 2B, scanning electron micrographs of RPE cells cultured without PSPA (control) and with 100 µg/ml PSPA (treated) for 2 days, showing the surface morphology. Scale bar: 10 μm. Fig. 2C, transmission electron micrographs of RPE cells cultured without PSPA (control) and with 100 µg/ml PSPA (treated) for 2 days, showing the ultrastructure of RPE cells (B, buds; Ch, chromosome; D, digitations; G, golgi apparatus; L, lysosome; Me, melanin granule; Mi, mitochondrion; Mv, microvilli; N, nucleus; Nn, nucleoli; Np, nuclear pores; P, phagocytic vacuoles; rER, rough endoplasmic reticulum, R, ribosomes). Scale bars: 5 μm for (i) and (j); 2 μm for (a), (c), (e), (d), and (f); 1 μm for (b), (g), and (h).

RPE cells cultured with and without 100 µg/ml PSPA both exhibited a flattened morphology growing in an even distribution cohesively after 2-day incubation (Fig. 2A(a) and (c)). Both groups predominantly exhibited a polygonal morphology in a mosaic arrangement, and some spindle-shaped cell phenotypes could also be seen (Fig. 2A(b) and (d)) after another 2-day incubation. Cells were more elongated than 2 days before. Moreover, there was no notable difference between the cells cultured with PSPA and the control group in cell growth morphology, adhesion, and distribution.

Colony-like cells with numerous short apical microvillus displayed both typical polygonal (Fig. 2B(a) and (d)) and spindle (Fig. 2B(b) and (e)) shapes. Cell–cell connection was also demonstrated in Fig. 2B(c): parts of two adjacent cells were confluent. Extensive extracellular matrix deposition at the edge of both groups of RPE cells was also observed by SEM microscopy. These matrix materials helped the RPE cells grow adhesively on the culture surface *in vitro*. RPE cells in mitosis are shown in Fig. 2B(f). Because the mitotic cycle of RPE cells is very short, the detection of mitotic figures within RPE cells cultured with 100 µg/ml PSPA proved that PSPA at this concentration showed no inhibition of cell proliferation.

Typical ultrastructures of cultured RPE (Fig. 2C) were observed. Melanin granules (melanosomes) (Fig. 2C(a) and (f)), characteristic of this kind of cell, were easily identified in both groups. Digitations and isolated, prominent buds (Fig. 2C(b) and (e)), as well as microvilli (Fig. 2C(j)), were detected on the cellular surface. Several organelles were found, for example mitochondria, rough endoplasmic reticulum, golgi apparatus, and lysosomes (Fig. 2C(b), (d), (e), (f), (g), and (h)). Ribosomes were prolific in both groups of cells (Fig. 2C(c), (g), and (h)). Phagocytic vacuoles (Fig. 2C(c) and (h)) were also observed in RPE cells cultured with PSPA, suggesting that PSPA did not affect the phagocytosis of RPE cells. Large-sized nuclei were balloon-shaped or tortuous (Fig. 2C(d) and (i)) and distributed erratically in the polygonal cells. Chromatin was dispersed and both group showed euchromatic nuclei. Sporadic nuclear pores (Fig. 2C(b), (e), and (h)) were observed and nucleoli (Fig. 2C(e) and (i)) were prominent. Although no dramatic ultrastructural changes were found between the two groups, the formation of chromosome was detected in Fig. 2C (j), indicating the mitosis of RPE cells cultured with PSPA. Thus TEM and SEM allowed visualization of similar structures (Fig. 2B(f)), which provided further evidence for our corollary that RPE cells cultured with PSPA have high proliferative potential. It was also in agreement with the higher OD values in the cytotoxicity study (Fig. 1).

### Effects of PSPA on the growth curve of RPE cells

**Fig. 3:**
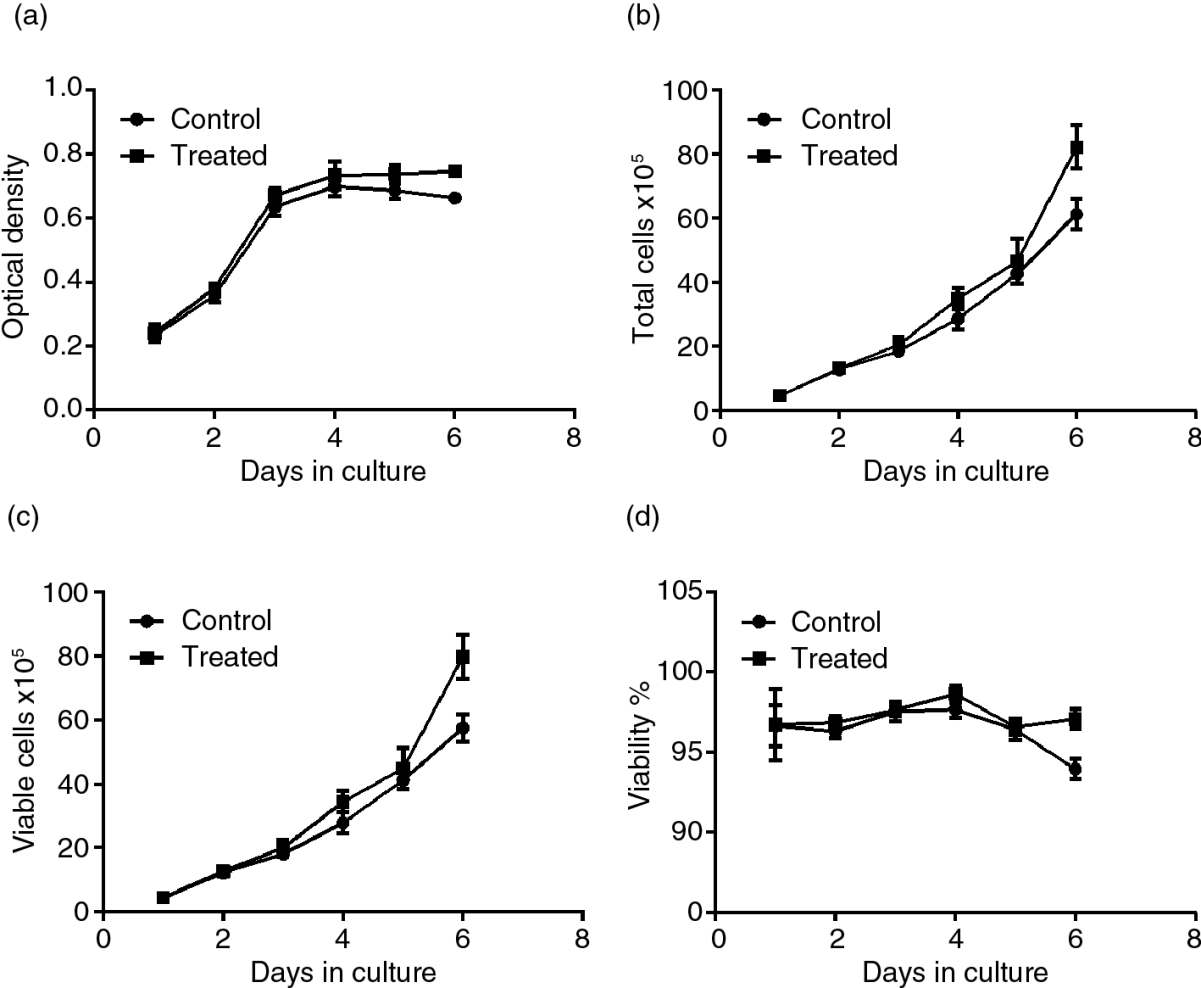
Effects of PSPA on RPE cell growth curve and viability. RPE cells without PSPA treatment were defined as the control; the effect of PSPA on the RPE cell growth curve and viability was evaluated at a dose of 100 µg/ml. (a) The growth curve of each group, expressed as optical density. Total cells, viable cells, and viability of each group were shown in b, c, and d, respectively (*n*=3).

To determine whether PSPA could change the growth rhythm of RPE cells, a line plot graph was constructed (Fig. 3a). The growth curves clearly showed that the RPE cells incubated with 100 µg/ml PSPA had a growth rhythm similar to the control group. However, cells cultured with PSPA had higher OD values compared to the control group from the fourth day of incubation (*p*=0.032). These results led to the assumption that PSPA might have effects on both cell division and survival, when given the cell population and individual cell as well. The potential of PSPA to promote individual RPE cell survival and longevity is supported by reports on the antiaging effects of antioxidants. Blueberry anthocyanin extracts were reported to suppress aging in replicatively senescent RPE cells by extending their lifespan, reducing the number of β-galactosidase-positive cells after subculturing, and inhibiting increases in intracellular reactive oxygen species (ROS) after visible light exposure (24). It was also demonstrated that quercetin (29), a natural plant-derived polyphenolic compound, was able to protect RPE cells from oxidative damage and cellular senescence *in vitro* in a dose-dependent manner. The maintenance of a specific number or density of healthy RPE cells *in vivo* is more important than turnover of the cell population for normal vision (30), whereas the reports on RPE cell survival have been limited. To our knowledge, our study is the first to demonstrate the protective effects of PSPA on RPE cell survival.

### Effects of PSPA on viability of RPE cells

Cell proliferation is, by definition, a balance between cell division and cell death (31). Thus, at first we examined the viability of RPE cells incubated with and without PSPA, which was independent of cell division, by counting the viable cells in all 2,000 events of each sample. As shown in Fig. 3b through 3d, roughly the same number of RPE cells with equal viability were seeded in each group, but after a 3-day incubation with PSPA, there were more total and viable cells in the PSPA group compared to the control. Both groups of RPE cells grew and proliferated very well. Total and viable cells in the control group exhibited 15.3-fold and 13.0-fold increases, respectively, and 17.9-fold and 18.0-fold increases, respectively, in the PSPA group at the end of the incubation period. The percentage of living cells was initially 96.69% and remained at greater than 96% for 4 days in both of the groups. At the 6-day point (Fig. 3d), the viabilities of the PSPA and control groups were 97.08 and 93.95%, respectively. A small but strongly statistically significant (*p*=0.0004) difference was shown, which suggested that PSPA could promote the survival and viability of RPE cells. This assay also provided further evidence that RPE cells treated with PSPA may have better platform growing status and activity, which was in line with the growth curve in Fig. 3a.

### Effects of PSPA on cell cycle of RPE cells

**Fig. 4:**
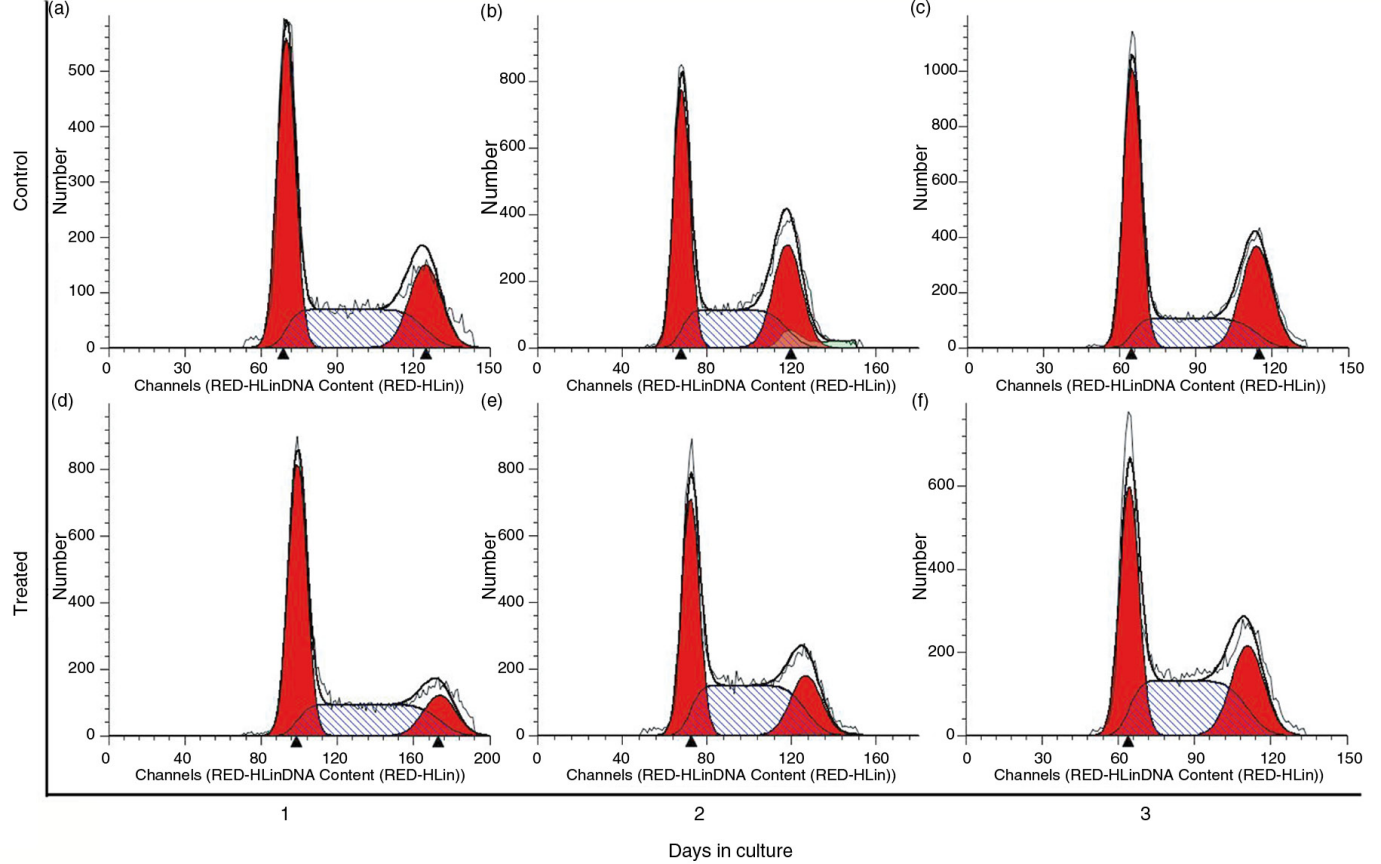
Cell cycle distribution of RPE cells without PSPA treatment (control) for 1~3 days (a through c), and RPE cells cultured with 100 µg/ml PSPA (treated) for 1~3 days (d through f).

Cell cycle analysis shed light upon the cells’ current cell cycle stage due to PSPA treatment. There were no differences in cycle distribution after a 1-day treatment (Fig. 4a and 4d), but after 2 days, PSPA incubation resulted in a striking decrease of G1/G0 stage cells (37.87±1.03% vs. 40.42±0.34%; *p=*0.0099), whereas the percentage of cells cycling in the S-phase increased significantly (44.71±3.24% vs. 31.14±0.48%; *p*=0.0009) (Fig. 4b and 4e). Moreover, obvious decline was also observed in the relative percentage of G2/M phase cells (17.43±2.46% vs. 28.43±0.47%; *p*=0.0007), and similar changes on the third day were recognized (Fig. 4c and 4f). Merging this phenomenon with the cellular morphology (Fig. 2) and viability (Fig. 3c and 3d) of RPE when treated with 100 µg/ml PSPA for 2 to 3 days, which had shown normal mitosis and more living cells with higher viability compared to the control group, it was clear that PSPA promoted DNA synthesis, which took place in the S-phase, rather than inducing cell cycle blockage. Cells undergo DNA synthesis in the S-phase, and DNA synthesis rates are determined as a proportion of the cell population in the S-phase or growth phase to indicate cell proliferation (32, 33). Moreover, an increase in the percentage of the RPE cell population in the S-phase of the cell cycle was also found in thrombin-induced RPE cell proliferation (34). It also explained why there were no dramatic increases in OD values until the fourth day of incubation with 100 µg/ml PSPA; from the cell growth curves seen in Fig. 3a, more active cell mitosis took place after a higher level of DNA synthesis.

### Effects of PSPA on Ki67 expression of RPE cells

**Fig. 5:**
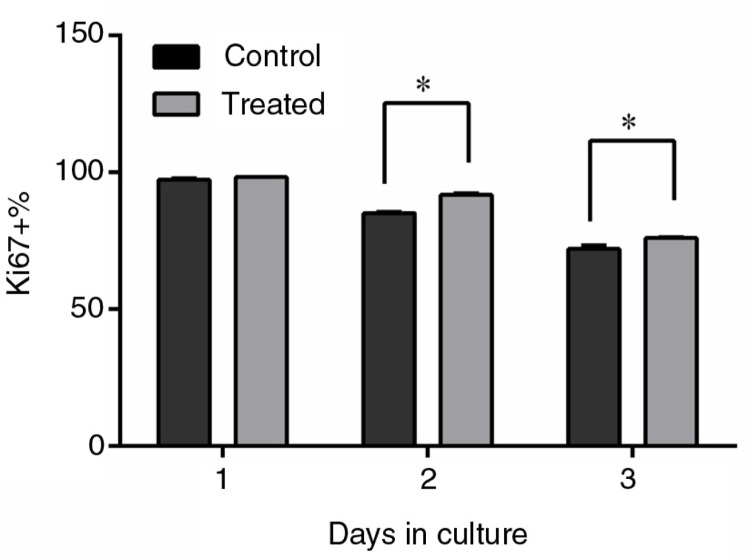
Ki67 expression of RPE cells treated without PSPA (control) and with 100 µg/ml PSPA (treated) for 1~3 days (*n*=3). *Statistical comparisons between PSPA-treated groups and controls were carried out on each day of incubation, and mean values were significantly different (*p*<0.05; unpaired Student *t*-test).

There are several markers expressed during cell proliferation, including Ki67, which is tightly associated with proliferation. Ki67 is a prototypic cell-cycle-related nuclear protein, expressed by proliferating cells in all active phases of the cell cycle (G1, S, G2, and M phases), but it is absent in the resting G0 phase (35). It was proven that Ki67 functions as a growth fraction marker, offering a good representation of proliferation status (36, 37). The Ki67 assay showed that the percentage of Ki67+ cells was higher when RPE cells were cultured with 100 µg/mL PSPA for 2 days (Fig. 5), which meant fewer cells in the G0 phase treated by PSPA. The percentages of Ki67+ cells were 85.13 and 91.87% for the control and PSPA group, respectively, after 2-day incubation, and 72.05 and 74.42% after 3-day incubation. It was noteworthy that the percentage of Ki67+ cells resulted in a dramatic decline during the incubation, which indicated that more and more cells would not undergo mitosis with the extension of incubation time. It was supposed that contact inhibition contributed to this decline in dividing cells. Hou (38) showed that ARPE-19 postconfluent cells rarely displayed Ki67 staining compared with subconfluent cells. A higher percentage of Ki67+ cells treated with PSPA indicated that more cells passed through the G1-phase to initiate DNA synthesis, which also contributed to the increase in S-phase cells. Ki67 assay data, together with cell cycle analysis data, revealed that incubation of cells in PSPA resulted in an increase of continuously cycling cells and promotion of DNA synthesis, boosting cell proliferation.

### Effects of PSPA on PI3K/MAPK expression of RPE cells

**Fig. 6:**
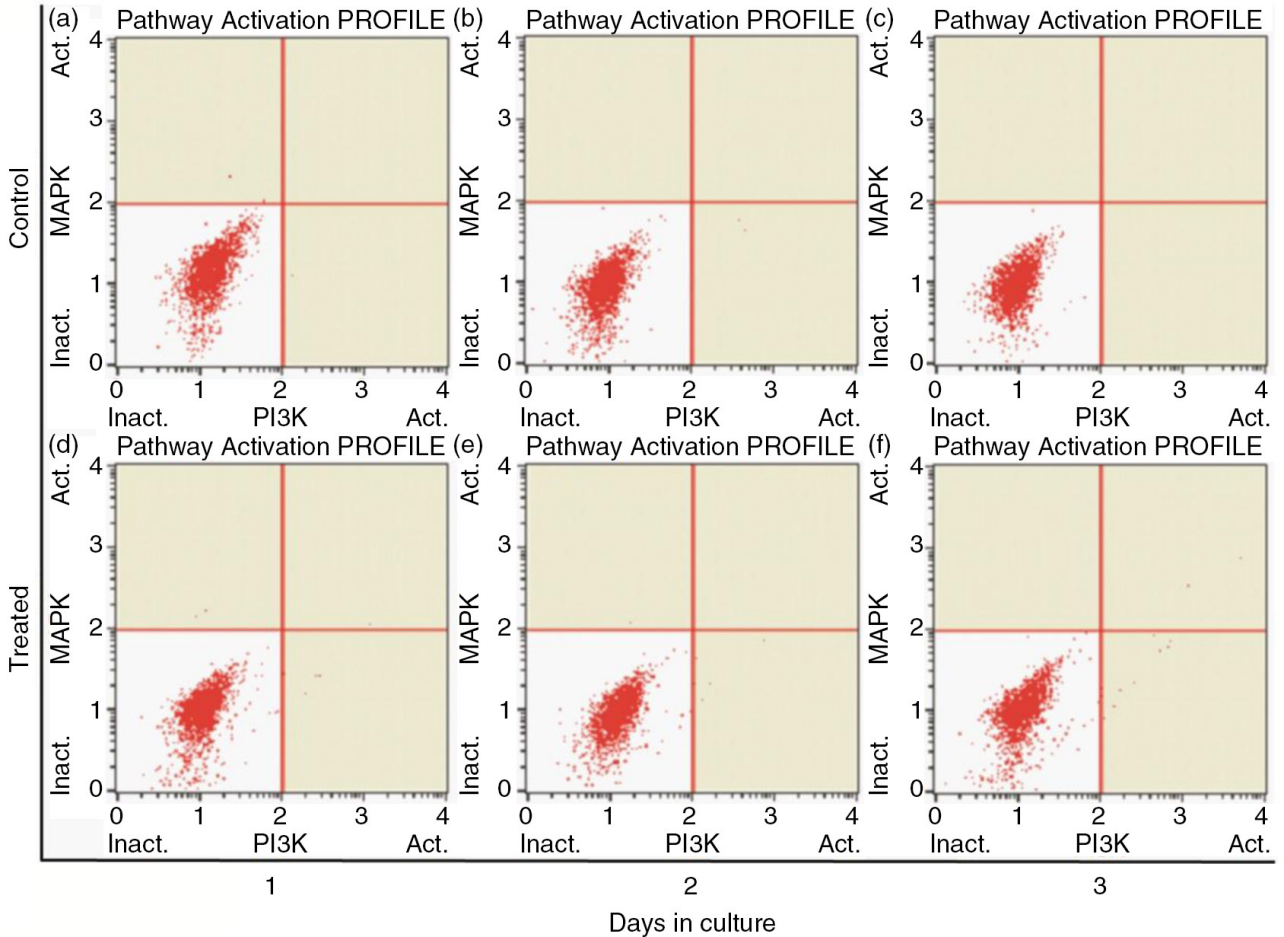
PI3K/MAPK expression of RPE cells treated without PSPA (control) and with 100 µg/ml PSPA (treated) for 1~3 days.

It has been reported that PI3K and MAPK signaling pathways are involved in the control of cell proliferation and cell survival (30, 39, 40). Moreover, PSPA was reported to play some of its functional roles through activating or enhancing the PI3K/Akt pathway (41, 42). In this study, we carried out the PI3K/MAPK dual pathway activation assay to determine whether the proliferation of RPE cells boosted by PSPA is mediated by Akt and/or ERK activation. As shown in Fig. 6, neither pAkt nor pERK was detected in either group in our experiments. It was supposed that there are other signaling pathways involved in RPE cell survival and division incubated by PSPA. The importance of integration of multiple intracellular signaling pathways in the regulation of RPE cell survival and longevity was also highlighted by Defoe (30). Parrales (34) showed that ERK activation was necessary but not sufficient for the induction of cyclin D1 expression and RPE proliferation. Hecquet (43) also demonstrated that MEK/ERK only participated in the signaling involved in cell growth, whereas the activation of the Ras/Raf-1 pathway was essential for fetal calf serum-induced RPE cell proliferation. PKC was reported to play an important role in the regulation of RPE cell proliferation (32). On the other hand, PSPA could activate other signaling pathways; for example, it significantly increased the phosphorylation of adenosine monophosphate-activated protein kinase (AMPK) in HepG2 hepatocytes, showing beneficial effects on prevention of obesity through mediating AMPK signaling pathways (44). Moreover, flavonoids have been reported to induce the expression of phase 2 proteins that function to enhance the cell's natural defenses against oxidative stress (3). Further study of kinases in multiple signaling pathways is necessary in order to elucidate the mechanism of PSPA effects on RPE cells.

## Conclusion

We found that PSPA maintained high cell viability, boosted DNA synthesis, and preserved a high percentage of continuously cycling cells to promote cell survival and division without changing cell morphology. These effects showed no involvement of phosphorylation of Akt and ERK. PSPA has a beneficial impact on the growth of RPE cells; the relationship between the structures of anthocyanins in PSPA and their functions on RPE cells merits further investigation. Experiments in this paper also laid the foundation for further research into the protective activities of PSPA against damage to retinal cells. We believe that PSPA is a promising candidate as a supplement for maintaining vision health.
